# Slipstreaming in Gravity Powered Sports: Application to Racing Strategy in Ski Cross

**DOI:** 10.3389/fphys.2018.01032

**Published:** 2018-07-31

**Authors:** Franz Konstantin Fuss

**Affiliations:** Smart Equipment Engineering and Wearable Technology Research Program, Centre for Design Innovation, Swinburne University of Technology, Melbourne, VIC, Australia

**Keywords:** sports engineering, aerodynamics, ski cross, slipstreaming, drafting, glide model, drag, lift

## Abstract

The principles of slipstreaming or drafting are very well known in muscle-powered sports, but unknown in gravity-powered sports. Typical examples of gravity-powered sports, where several athletes are racing against each other, are ski-cross and snowboard-cross. The aim of this research is to investigate the effectiveness and practical applicability of slipstreaming in ski-cross. A glide model consisting of leading and trailing skiers was developed and used with existing aerodynamic drag and lift data sets from wind tunnel tests. Different scenarios were tested as to their effect on slipstreaming, such as variation of speed, skiers' mass, slope angle, air density, and racing posture (high/low tucked position). The higher the trailing skier's inertial force and acceleration is compared to the leading one, the quicker the trailing skier can catch up. Making more ground up on the racing track is related to higher speed, less body mass (of both skiers), flatter slope angle, denser air, and higher racing posture (high tucked position of both skiers). The glide model presented in this research can be used in the future for testing of slope track design, provided that precise dimensions of terrain features are available.

## Introduction

Slipstreaming or drafting is a commonly used strategy in sports, specifically in cycling (Barry et al., 2014, 2015), speed skating (Rundell, 1996), running (Pitcher, 2009), wheelchair racing, and other sports. These sports disciplines, however, are muscle-powered, where slipstreaming reduces energetic demands. There is no single study on gravity-powered sports, probably because there is often only one athlete or team on the track rather than directly competing against each other. Classical gravity-powered sports are bobsleigh (after the start phase), luge (after the start phase), skeleton, alpine skiing, ski jumping, and snowboarding. However, in 2006 and 2010 respectively, snowboard-cross and ski-cross became Olympic disciplines, where 4–6 athletes are racing against each other on the same track. Although there is no judged component, these disciplines are still considered freestyle because of terrain features typical for freestyle. Baggy and fluttering clothing is another freestyle feature, actually prescribed by the ski cross rules (FIS, 2017, rule 4511.6 Suit Measurement). Yet, as in alpine skiing, speed is crucial and the first athlete that crosses the finish line wins, which in turn requires obeying aerodynamics principles.

Slipstreaming is governed by interference drag (Hoerner, 1965). When two bluff bodies are aligned in series in the free airstream, the drag force on the trailing body decreases as the bodies get closer. There is also an effect on the leading body with a slight reduction of drag.

Fuss (2011) investigated the drag forces on trailing and leading skiers with wind tunnel tests, and the results followed the expected principles of bluff-body interference drag, as outlined by Hoerner (1965). It is, however, unknown, whether these results are practically effective in gravity-powered sports. In contrast to muscle-powered sports, slipstreaming in gravity-powered sports is not applicable to saving the athlete's muscle power (required for propulsion) but should rather influence the trailing skier by catching up with the leading one, i.e., closing the distance between two athletes racing downhill back to back. This is all the more important in ski- and snowboard cross, as often only 10–20 cm determine a win.

The aim of this study is to derive a strategy for slipstreaming in ski-cross from a glide model, and provide advice and practical recommendations for athletes.

## Methodology

The method consists of the following procedures:

1. establishing functions of aerodynamic drag and lift with respect to the distance between leading and trailing skiers from existing data sets (Fuss, 2011);
2. develop a glide model that returns speed and distance glided; and
3. testing the numerical version of the model with two skiers and different parameters in order to understand the dynamics of slipstreaming (e.g., is slipstreaming more efficient at a high or low tucked position?).

In order to assess how the distance between two skiers changes when racing the following pre-requisites are required:

1. aerodynamic drag and lift areas (*Ad* and *Al*) of the trailing skier as a function of distance *D* between two skiers;
2. aerodynamic drag and lift areas of the leading skier as a function of distance *D* between two skiers;
3. a mathematical glide model.

*Ad* and *Al* are the drag and lift coefficients (*C*_*D*_ and *C*_*L*_) multiplied by the projected areas *A*, and calculated from

(1) $$\begin{array}{rcl} F_{D} & = & {\frac{\rho^{}{C_{D}}^{}A^{}v^{2}}{2}}^{}\;\to^{}\;C_{D}A=Ad=\frac{2F_{D}}{\rho v^{2}} \end{array}$$

(2) $$\begin{array}{rcl} F_{L} & = & {\frac{\rho^{}{C_{L}}^{}A^{}v^{2}}{2}}^{}\;\to^{}\;C_{L}A=Al=\frac{2F_{L}}{\rho v^{2}} \end{array}$$

where *F*_*D*_ and *F*_*L*_ are drag and lift forces, *v* is the free-stream velocity, and ρ is the air density.

For establishing the functions of *Ad* and *Al* against distance *D*, the data of Fuss (2011) were fit with different functions. In addition to the data of Fuss (2011), extreme data were included in the dataset that helped establish the correct asymptotic values of the fit functions in absence of measurement data. These extreme values were:

- leading skier: at *D* = 0, *Ad* = *Ad*_max_ and *Al* = *Al*_max_, as the interference drag and lift on the leading body returns to the original single-body drag and lift if the distance *D* closes to zero. This is only of theoretical importance in skiing, however, essential for correct modeling. When plotting *D* on a logarithmic scale, then *Ad* and *Al* asymptote to *Ad*_max_ and *Al*_max_ as *D* approaches 0.
- trailing skier: at *D* = 0, *Ad* = *0* and *Al* = *0*, as the trailing body does not experience any drag after having merged with the leading body.
- trailing and leading skiers: at *D* = ∞, *Ad* = *Ad*_max_ and *Al* = *Al*_max_, as the interference drag vanishes at large *D*; practically, no interference drag is expected at *D* = 100 m, which means that the asymptotic value should have been reached at *D* = 100 m.

**Figure 1:**
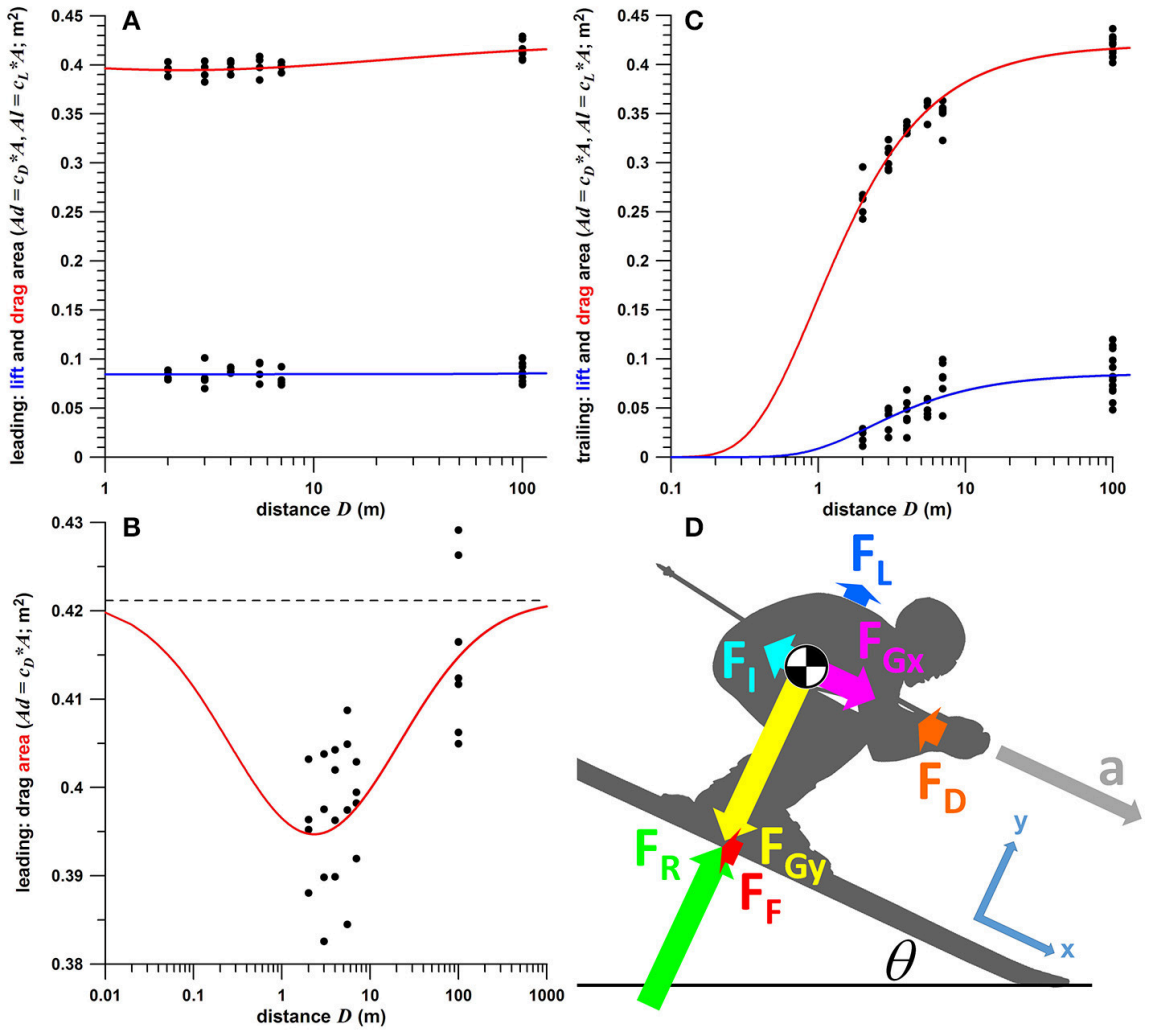
**(A–C)** Drag and lift area, *Ad* and *Al* (coefficient of drag and lift times projected area) against distance between the two skiers (data from Fuss, 2011) including fit functions (red: drag area, blue: lift area); **(A)** leading skier; **(B)** enlarged graph of drag area of leading skier; **(C)** trailing skier; **(D)** free body diagram of a skier (the coordinate system of which is aligned to the slope), including vectors of acceleration (*a*) and forces (*F*_*D*_ and *F*_*L*_ are drag and lift forces; *F*_*Gx*_ and *F*_*Gy*_ are the *x*- and *y*-components of the gravitational force; *F*_*R*_ is the ground reaction force acting on the skis; *F*_*F*_ is the friction force acting on the skis; and *F*_*I*_ is the inertial force); the relative size of the force vectors is true for a slope angle (θ) of 25°, a mass of 100 kg (body plus gear), and a speed of 100 kph; 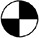 = center of mass.

The following fit functions were used:

- *Ad* of leading skier: fitted by a negative Gaussian function of the decadic logarithm of *D* (*y* = *a*–*b*e^(logx−*c*)/*d*^; where *a* = *Ad*_max_; Figure 1A). The Gaussian function also provides identical asymptotic *Ad* values for small and large distances (decadic logarithm of *D;* Figure 1B);
- *Al* of leading skier: fitted by an average fit (constant *Al*) as this parameter was not affected by the distance *D*. (Figure 1A);
- *Ad* and *Al* of trailing skier: exponential functions of the form *y* = *a*+*b*e^−c/*x*^ (where *a* = 0), as drag and lift asymptote to their maximum values at large distances and to zero at very small distances (Figure 1C).

Care was taken that the asymptotic *Ad* and *Al* values were the same for both leading and trailing skiers. This was required for the first modeling step. The data shown in Figure 1 refer to distances of *D* = 2, 3, 4, 5.5, and 7 m. The unequal spacing of these data (1 m and 1.5 m increments) does not substantially influence the fit results. When taking the *Ad* and *Al* data of *D* = 5.5 and using them at hypothetical *D* of 5 and 6 m (instead of 5.5 m), then the fit deviates on average only by 0.09% of *Ad*_max_ of the trailing skier, by 0.40% of *Al*_max_ of the trailing skier, and by 0.03% of *Ad*_max_ of the leading skier.

The glide model was based on the free-body diagram of a skier gliding downhill, and all the forces acting on it (Figure 1D). Although glide models were already developed by Luethi and Denoth (1987; numerical), Broker (1991; inaccessible), and Nørstrud (2008a; 2008b with lengthy derivations), a straightforward analytical solution is presented subsequently.

The force equilibriums in *x*- and *y*-directions (Figure 1D) are:

(3) $$\begin{array}{rcl} F_{Gy} & = & F_{R}+F_{L} \end{array}$$

(4) $$\begin{array}{rcl} F_{Gx} & = & F_{I}+F_{F}+F_{D} \end{array}$$

where the *x*–coordinate of the coordinates system is parallel to the slope and pointing downhill and the *y*–coordinate is perpendicular to the slope pointing upwards and forwards; *F*_*Gx*_ and *F*_*Gy*_ are the *x*- and *y*-components of the gravitational force (skier plus gear); *F*_*R*_ is the ground reaction force acting on the skis; *F*_*F*_ is the friction force (uphill) acting on the skis; *F*_*I*_ is the inertial force (uphill) opposite to the acceleration vector (downhill) acting on the skier; and *F*_*D*_ and *F*_*L*_ are drag- and lift-forces, acting on the skier in uphill and upward/forward direction, respectively.

Solving Equation (3) for *F*_*R*_, and substituting $\mu^{}F_{R}\;=\;\mu^{}\left(F_{Gy}-F_{L}\right)$ for *F*_*F*_ in Equation (4) yields

(5) $$\begin{array}{r} F_{I}=F_{Gx}-\mu^{}F_{Gy}+\mu^{}F_{L}-F_{D} \end{array}$$

where μ is the kinetic coefficient of friction, resulting in

(6) $$\begin{array}{r} a^{}\;m=g^{}\;m^{}\sin\theta -\mu^{}\;g^{}\;m^{}\cos\theta +\mu^{}F_{L}-F_{D} \end{array}$$

where *a* is the acceleration of the skier, *m* is the mass of skier plus gear, θ is the slope angle (Figure 1D; θ is positive), and *g* is the gravitational acceleration. The differential equation to be solved is

$$\begin{array}{rcl} \frac{\text{d}v}{\text{d}t} & = & \left(g^{}\sin\theta -\mu^{}g^{}\cos\theta \right)+\frac{\mu }{m}F_{L}-\frac{1}{m}F_{D} \end{array}$$

(7) $$\begin{array}{rc} = & c_{1}+\frac{\mu }{m}\frac{\rho^{}Al}{2}v^{2}-\frac{1}{m}\frac{\rho^{}Ad}{2}v^{2}=c_{1}+c_{3}v^{2}-c_{2}v^{2} \end{array}$$

where $c_{1}=g^{}\sin\theta -\mu^{}g^{}\cos\theta$, $c_{2}=0.5\rho^{}Ad/m$, and $c_{3}=0.5\rho^{}Al\mu /m$.

After rearranging and defining *c*_4_ = *c*_2_−*c*_3_ (as *Ad*>μ*Al*),

(8) $$\begin{array}{r} \frac{\text{d}v}{\text{d}t}=c_{1}-c_{4}v^{2} \end{array}$$

Solving for d*t*

(9) $$\begin{array}{r} \text{d}t=\frac{1}{c_{1}-c_{4}v^{2}}dv \end{array}$$

and integrating both sides

(10) $$\begin{array}{r} t_{1}-t_{0}={\overset{v_{t}}{\underset{v_{0}}{\int }}\frac{1}{c_{1}-c_{4}v^{2}}}^{}dv \end{array}$$

where *t*_0_ = 0. Solving the integral yields

(11) $$\begin{array}{r} t={\left[\frac{\tanh^{-1}\left(v\sqrt{\frac{c_{4}}{c_{1}}}\right)}{\sqrt{c_{1}c_{4}}}\right]}_{v_{0}}^{v_{t}} \end{array}$$

and

(12) $$\begin{array}{r} t\sqrt{c_{1}c_{4}}=\tanh^{-1}\left(v_{t}\sqrt{\frac{c_{4}}{c_{1}}}\right)-\tanh^{-1}\left(v_{0}\sqrt{\frac{c_{4}}{c_{1}}}\right) \end{array}$$

Solving for *v*_*t*_ yields

(13) $$\begin{array}{r} v_{t}={\sqrt{\frac{c_{1}}{c_{4}}}}^{}\tanh\left[\tanh^{-1}\left(v_{0}\sqrt{\frac{c_{4}}{c_{1}}}\right)+t\sqrt{c_{1}c_{4}}\right] \end{array}$$

i.e., the velocity as a function of time.

Simplifying Equation (13) by defining three further constants, $c_{5}=\tanh^{-1}\left(v_{0}\sqrt{\frac{c_{4}}{c_{1}}}\right)$, $c_{6}=\sqrt{c_{1}c_{4}}$, and $c_{7}=\sqrt{\frac{c_{1}}{c_{4}}}$, yields

(14) $$\begin{array}{r} v_{t}={c_{7}}^{}\tanh\left(c_{5}+c_{6}t\right) \end{array}$$

*c*_7_ constitutes the terminal velocity *v*_*term*_ where *a* = 0 and consequently *F*_*I*_ = 0, and *F*_*Gx*_ = *F*_*F*_+*F*_*D*_:

$$\begin{array}{rcl} c_{7} & = & v_{term}=\sqrt{\frac{c_{1}}{c_{4}}}=\sqrt{\frac{gsin\theta -\mu g\cos\theta }{c_{2}-c_{3}}} \end{array}$$

(15) $$\begin{array}{rc} = & \sqrt{\left(\frac{2mg}{\rho }\right)\left(\frac{sin\theta -\mu \cos\theta }{Ad-\mu Al}\right)} \end{array}$$

Reducing Equation (15) to large variables, by removing common constants and small variables (i.e., *Al*, as *Ad* ≈ 60μ*Al*), yields

(16) $$\begin{array}{r} v_{term}\propto \sqrt{\frac{m}{Ad}} \end{array}$$

where the right part of Equation (16) is equivalent to the “*anthropometric code number*” by Luethi and Denoth (1987; who used *mg* instead of *m*), explaining why heavier and smaller skiers are faster. The practical application of Equation (16) is, what every head coach should do, namely calculate this ratio, and compare and rank the team members. This method is also essential for drafting new team members. The data required for this ration are (1) the mass of the skier plus gear, and (2) *Ad* and *Al* either from wind tunnel tests or from glide tests by recording the speed with a ski speed meter (e.g., vLink™ by Advanced Racing Computers, Salt Lake City, UT, USA; Kirby, 2009). The data obtained from the speed meter at a realistic speed for different and defined tucked positions is the velocity as a function of time, which can be fitted with the function given in Equation (14), to obtain *Ad*, but also an estimate of *Al* and μ, if realistic fit boundaries are selected.

Integrating Equation (14) for calculating the displacement *x* on the slope, for initial conditions of *t*_0_ = 0 and *x*_0_ = 0:

(17) $$\begin{array}{r} x_{t}={c_{7}}^{}{\overset{t_{1}}{\underset{t_{0}}{\int }}\tanh\left(c_{5}+c_{6}t\right)}^{}dt \end{array}$$

yields

(18) $$\begin{array}{r} x_{t}={\frac{c_{7}}{c_{6}}}^{}\left\{\ln\;\left[\cosh\left(c_{5}+c_{6}t\right)\right]-\ln\;\left[\cosh\left(c_{5}\right)\right]\right\} \end{array}$$

where ln denotes the natural logarithm. Solving Equation (18) for *t* yields:

(19) $$\begin{array}{r} t_{x}=\frac{\cosh^{-1}\left\{e^{\frac{c_{6}}{c_{7}}x+\ln\;\left[\cosh\left(c_{5}\right)\right]}\right\}-c_{5}}{c_{6}} \end{array}$$

There are two boundary conditions related to the derivation of the glide model equations. From Equation (11) it becomes evident that the constants *c*_1_ and *c*_4_ must be positive. Constant *c*_4_ is larger than 0 by definition, as *Ad* > μ *Al*. Solving $c_{1}=g^{}\sin\theta -\mu^{}g^{}\cos\theta$ for θ reveals that θ ≥ tan^−1^μ for *c*_1_ ≥ 0. If μ = 0.05, then the critical slope angle would be 0.04996 rad which equals 2.862°. Therefore, how would slope angles smaller than 2.862° influence the glide model, if *c*_1_ were smaller than 0, and so were the arguments of the square roots of constants *c*_5_, *c*_6_, and *c*_7_? The answer is given by the equation of *c*_5_: the argument of the inverse hyperbolic tangent function has to be smaller than 1 (2nd boundary condition). This, in turn, implies that *v*_0_ cannot be greater than *c*_7_. If *v*_0_ = *c*_7_, then the argument of the inverse hyperbolic tangent function is exactly 1. This further implies that, if $v_{0}\sqrt{\frac{c_{4}}{c_{1}}}\leq 1$, then $\sqrt{\frac{c_{1}}{c_{4}}}\geq v_{0}$ and *v*_*term*_ ≥ *v*_0_. Consequently, if *c*_1_ = 0, then *v*_*term*_ = 0, which implies that *v*_0_ has to be zero as well, in order to keep *c*_5_ real.

The condition of *c*_1_ ≥ 0 and its associated slope angles of θ ≥ tan^−1^μ are irrelevant, as even angles of θ > tan^−1^μ can still be outside the gravity-powered domain. This means that at *c*_1_ = 0 gravity can no longer accelerate the skier, as the forces accounting for non-conservative energy (drag and friction) decelerate the skier and therefore outweigh the effect of gravity. As such, there must exist a critical slope angle at which decelerating forces are in equilibrium with gravity, resulting in zero acceleration on an inclined slope. The critical slope angle, θ_*crit*_, can be derived from Equation (6), by setting the acceleration *a* to zero. Solving for θ yields

(20) $$\begin{array}{r} \theta_{crit}=\sin^{-1}\left(\frac{\frac{c_{4}}{g}v^{2}+\sqrt{-\frac{{c_{4}}^{2}\mu^{2}}{g^{2}}v^{4}+\mu^{2}+\mu^{4}}}{\mu^{2}+1}\right) \end{array}$$

At θ_*crit*_, *v*_*term*_ ≡ *v*_0_, which is evident as there is no acceleration at the boundary of the gravity-powered domain, which fulfills the basic condition of *v*_*term*_ ≥ *v*_0_ (argument of the inverse hyperbolic tangent function ≤ 1). As θ_*crit*_ > tan^−1^μ (unless *Al* or μ are excessively and unrealistically high), *c*_1_ > 0.

For the glide model, the following constants were pre-defined: initial velocities, *v*_0L_ and *v*_0T_, of leading and trailing skiers, respectively; initial displacements *x*_0L_ and *x*_0T_ (where *x*_0T_ = 0, and *x*_0L_ = *D*_0_, i.e., the initial distance between the two skiers); body masses *m*_*L*_ and *m*_*T*_; θ, μ, ρ (depending on altitude and air temperature of the slope) and *g*. The velocities, *v*_*L*_ and *v*_*T*_, and displacements, *x*_*L*_ and *x*_*T*_, were calculated numerically for each time step. *Ad* and *Al* (of leading and trailing skiers), defined as per fit functions of *D* (Figure 1), were updated after each time step. *D* is determined from *x*_*L*_–*x*_*T*_, and the ground made up by the trailing skier, Δ*x*, equals *D*_0_-*D*. Δ*x* was determined for *D*_0_ ranging from 2 to 20 m, for glide distances from 5 to 200 m. Subsequently, the pre-defined constants were varied to understand different glide scenarios.

## Results

**Figure 2:**
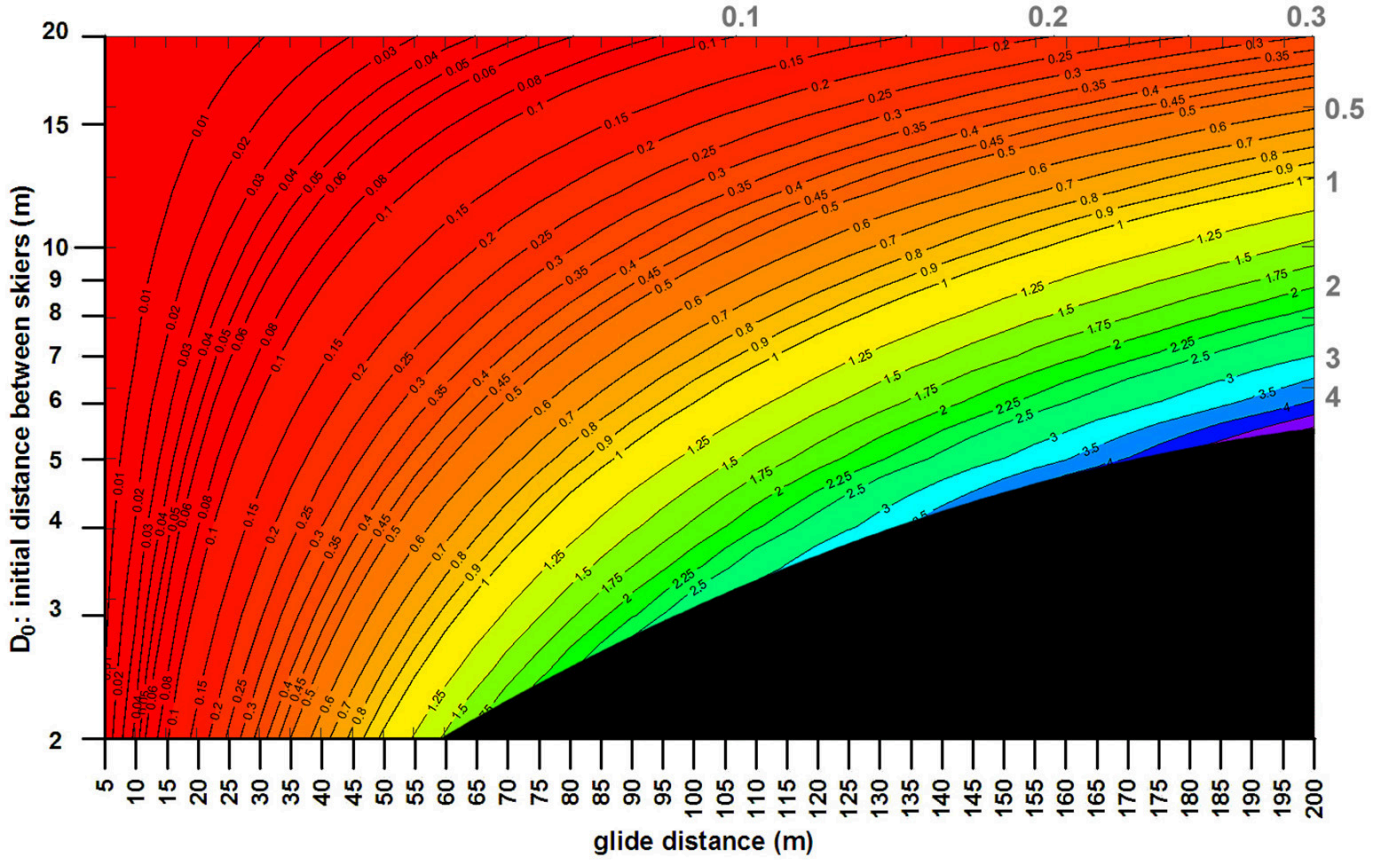
Initial distance (*D*_0_) between the two skiers against the glide distance; the contour lines (and the gray values at the top and right side of the plot) correspond to the ground made up (in meters) across the glide distance. The black triangle at the bottom right corner corresponds to unfeasible ground made up, i.e., the minimum distance between the skiers at the end of the glide distance is confined to 0.5 m. In this contour plot, the conditions for both skiers were as follows: mass (body plus gear) = 90 kg, kinetic friction coefficient between ski and snow = 0.05, initial speed at the beginning of the glide = 70 kph; slope angle = 20°; air density = 1.2 kg/m^3^ (e.g., at 1,000 m altitude, −12.3°C, and humidity < 60%). At these conditions, if the initial distance between the two skiers is 6 m at the beginning of the glide, then the ground made up after a 145 m glide is 2 m (contour), with a final distance of 4 m between the two skiers.

Figure 2 shows the basic principle of slipstreaming: the smaller *D* and the longer the glide distance, the more ground can be made up (Δ*x*) by closing the distance *D*. In Figure 2, the two skiers were at a high tuck position with identical conditions (*m* = 90 kg, μ = 0.05, *v*_0_ = 70kph; θ = 20°; ρ = 1.2 kg/m^3^). For example, at a glide distance of 100 m and *D*_0_ = 4 m, Δ*x* equals 1.8 m. This value and its associated conditions will subsequently be referred to as the “*reference condition*,” which further changing conditions will be compared to.

Δ*x* is only slightly dependent on speed. Compared to the reference condition and its Δ*x* of 1.8 m, Δ*x* = 1.88 m at 90 kph and 1.69 m at 50 kph. If *D*_0_ = 10 m, Δ*x* = 0.46 m at 90 kph and 0.41 m at 50 kph. Slipstreaming is therefore slightly more effective at higher speeds.

If the velocities of the two skiers are different, making up ground depends on the speed differential. Compared to the reference condition, if the trailing skier is 10 kph faster (80 kph), only a 14.9 m glide distance over 0.65 s is required for catching up by 1.8 m (compared to 100 m or 4.28 s at an initial speed of 70 kph); it would take 15.3 m or 0.67 s without slipstreaming if the two skiers were racing side by side (not too close, though, as otherwise interference drag arises). If the trailing skier is only 1 kph slower (69 kph), then the distance between the two skiers will increase, in spite of slipstreaming, from 4 m to a maximum of 4.21 m after a glide of 31 m (or to 4.39 m without slipstreaming), and then return to 4 m after a 65.3 m glide (or further increase to 4.7 m without slipstreaming). Trailing at a speed of 65 kph, e.g., after having been overtaken by the leading skier, and entering the slipstream at 4 m distance, the distance between the two skiers will increase from 4 m to a maximum of 8.51 m after a glide of 181.7 m (and to 10.84 m without slipstreaming).

Δ*x* is dependent on the mass of the skiers, i.e., the lighter the pair of skiers, the more ground the trailing skier makes up over the same glide distance. Compared to the reference condition, Δ*x* = 2.34 m if the mass of both skiers is 70 kg each, and 1.47 m at 110 kg.

If the masses of the two skiers are different, then Equation (16) explains why a trailing skier with less mass than the leading one is disadvantaged. Compared to the reference condition, if the trailing skier is 10 kg lighter (80 kg) or heavier (100 kg), Δ*x* is 0.61 m and 2.75 m, respectively. If the mass of the trailing skier is 75.7 kg at the same conditions, there is no gain from slipstreaming (< 1.5 mm at 100 m). Beyond this critical mass, Δ*x* is negative and *D* increases. This fact, however, should not discourage skiers from slipstreaming, as the loss in distance is worse without slipstreaming. At *D*_0_ = 10 m, the critical mass of the trailing skier increases to 86.2 kg, only 3.8 kg less than the mass of the leading skier.

The friction coefficient μ has negligible influence on Δ*x*. Compared to the reference condition, changing μ by ± 0.025 results in a change of Δ*x* by ∓ 0.015 m.

The air density ρ changes with altitude, temperature and humidity, all of which are negatively correlated with ρ. ρ and Δ*x* show the same behavior: less dense air results in smaller Δ*x*; at small glide distances, the relative changes of ρ and Δ*x* are similar; at longer glide distances, the relative change of Δ*x* is slightly smaller than the one of ρ.

The slope angle θ is negatively correlated to Δ*x*: the steeper θ, the smaller is Δ*x*. At the reference condition, Δ*x* = 1.8 m; changing the slope angle to 10° and 30° results in Δ*x* of 1.93 and 1.73 m.

Changing the racing position from high to low tuck decreases the aerodynamic drag (roughly by 40% on average; Fuss, 2011). Reducing *Ad* to 60% for both skiers at the reference condition shortens Δ*x* to approximately the same percentage (59% at short glide distances and 58% at 100 m). The lower the tuck position, the smaller is Δ*x*. The influence of *Al* on Δ*x* is negligible. When increasing *Al* by 20%, Δ*x* decreases by < 1% (at the reference condition).

Once the distance between the leading and trailing skiers has closed to an amount that requires the trailing skier to overtake the leading one, then the following questions arise:

- how does the suddenly increasing drag force, when breaking out of the slipstream, affect the trailing skier;
- is overtaking still possible under these circumstances; and, if yes,
- how long does it take to overtake the leading skier?

In principle, the tailing skier is always faster than the leading one, if the distance between the two skiers has decreased. The suddenly increasing drag force merely affects the acceleration of the trailing skier, whereas his/her velocity still increases due to gravity. The trailing skier should slipstream as long as practically possible and as long as he/she can overtake safely without endangering the leading skier.

For example, considering the reference condition, the trailing skier wants to break out of the slipstream at *D* = 1.5 m. This would happen after a glide distance of 116.6 m and a glide time of 4.88 s. The trailing skier then experiences the drag and lift he/she would without slipstreaming and will overtake the leading skier after a further glide of 35 m and 1.24 s. At that moment, the overtaking skier is 1.1 m/s faster (28.73 m/s).

Decreasing the slope angle (to 10°), or increasing the initial speed (to 90 kph), or increasing the skiers' mass (to 100 kg), does not substantially change the glide distance (112.6–122.8 m), nor the glide time (4.1–5.5 s), the further glide distance after leaving the slipstream (33–36.7 m), the further glide time (1.1–1.5 s) or the speed differential (0.89–1.24 m/s).

If the drag area of the reference condition is reduced to 60%, then the glide distance and time required for *D* = 1.5 m changes to 150.1 m and 5.83 s, and the glide distance and time required for overtaking changes to 44 m and 1.36 s. The speed differential at the time of overtaking is 1 m/s (at a total speed of 33.1 m/s). Therefore, slipstreaming enables faster overtaking.

## Discussion

The explanation for the principles outlined in the Results section is found in Equation (5), rewritten as the ratio of:

(21) $$\begin{array}{r} \frac{{\left(F_{I}\right)}_{trailing}}{{\left(F_{I}\right)}_{leading}}=\frac{{\left(F_{Gx}-\mu^{}F_{Gy}\right)}_{trailing}-{\left(F_{D}-\mu^{}F_{L}\right)}_{trailing}}{{\left(F_{Gx}-\mu^{}F_{Gy}\right)}_{leading}-{\left(F_{D}-\mu^{}F_{L}\right)}_{leading}} \end{array}$$

separated in gravitational and aerodynamic contribution on the right side.

The higher this ratio, the larger the acceleration of the trailing skier compared to the leading one, and the quicker the trailing skier can catch up. There is always a difference in *Al* and *Ad*, i.e., in *F*_*L*_ and *F*_*D*_. Note that μ*F*_*L*_ < < *F*_*D*_ and that *F*_*D*_ is subtracted. Thus, if less drag is subtracted, the numerator increases and the ratio is greater than unity. Decreasing the mass of both skiers reduces the influence of the gravitational force such that the ratio, now dominated by the drag force, increases. Decreasing the slope angle reduces *F*_*Gx*_, which in turn outweighs the increase of *F*_*Gy*_ (as multiplied by μ) so that the effect is the same as decreasing the mass. Opening up the tuck position subtracts more aerodynamic contribution from the same gravitational one (on either side of the division sign), which increases the ratio.

Slipstreaming has two decisive advantages: the slipstreaming athlete

- can catch up quicker with the leading one, or at least reduce speed loss caused by aerodynamic drag; and
- is able to save muscle energy.

The latter effect becomes apparent when comparing the advantage of slipstreaming to a reduction of aerodynamic drag by changing the body position without slipstreaming.

At the reference condition, making ground up of 1.8 m (100 m glide distance, 4.28 s glide time) has the same effect as reducing *Ad* and *Al* to 83.14% (4.3 s, 100 m) at a negligible 0.16% longer glide time. This can only be done by adopting a deeper tucked position, which consumes more muscle energy and also prevents the athlete from reacting quicker.

Putting this into an energy perspective, in terms of energy lost to drag and energy produced by the quadriceps muscle for maintaining the tucked position, results in the following data:

Slipstreaming: the energy of the trailing skier lost to drag (integral of drag force with gliding distance) amounts to 10.52 kJ; the increase in kinetic energy (initial speed: 19.4 m/s, final speed 26.9 m/s) equals 15.60 kJ.

Without slipstreaming, and instead reducing *Ad* and *Al* to 83.14%: the energy lost to drag is 11.61 kJ (i.e., 10.35% more energy is lost compared to the slipstreaming case); the increase in kinetic energy equals 14.57 kJ (i.e., the energy gain is 6.63% less than during slipstreaming).

In terms of comparing the muscle energies required for maintaining the two different body positions (higher tuck with slipstreaming and lower without), the relationship between energy expenditure of (isometric) contraction and muscle force produced must be known. According to Ortega et al. (2015), the isometric cost (unit: J) is a linear function of the force-time integral of the muscle. Based on a high tuck position (the aerodynamic data of which are shown in Figures 1A–C) and a lower tuck position (with an *Ad* of 83.14% of the high tuck position), the summation COM (center of mass) of all body segments above the knee was calculated (based on the body segment data of Drillis and Contini, 1966; for a body height of 1.8 m and body+gear mass of 90 kg). The moments of *F*_*R*_ and *F*_*F*_ about the knee joint (minus the moments produced by the gravitational force of shanks, feet, boots and skis) resulted in 32.5 Nm and 62.4 Nm, respectively. This means that the knee moment in the lower tucked position was 1.92 times higher than in the high tucked one (slipstreaming condition), i.e., 92.20% higher. This relative relationship of the two knee moments does not mean that muscle force is also roughly 2 times higher, considering that that reducing the drag area requires a higher knee flexion angle (increase from 65 to 88°), which in turn decreases the (internal) moment arm of the patellar ligament as well as the mechanical advantage of the patellofemoral joint. Both factors would increase the muscle force (quadriceps); the muscle force is therefore expected to be greater than just 2 times the one at the higher tucked position. Neglecting the 0.16% longer glide time (for calculating the force-time integral), the isometric cost, and therefore the energy expenditure, of the quadriceps is estimated to be at least 2 times higher without slipstreaming compared to the slipstreaming condition.

These energy comparisons lead to recommendations for racing strategies in ski cross:

1. General recommendations not necessarily confined to slipstreaming:
  Tradeoff between aerodynamics and muscle energy expenditure:
  From an aerodynamic point of view, the lowest tuck is the best choice. However, apart from the inability to react quickly to changing conditions, the muscle energy expenditure is higher, specifically for the quadriceps muscle.
  In general, the lower the tucked position, the smaller is the drag area, so that less energy is lost to drag. Nevertheless, athletes cannot react that quickly to changing terrain features out of a deep tuck, and the risk of crashing is greater. This is why athletes prefer a higher tuck taking into account more energy lost to drag.
2. Specific recommendations for slipstreaming:
  Slipstreaming does have an advantage. Therefore, slipstreaming is recommended whenever feasible, as slipstreaming (a) increases the speed of the trailing skier relative to the leading one, or, at least, (b), minimizes the speed loss relative to the leading one.

In addition to this basic recommendation, there are situations when slipstreaming is more beneficial than in others:

The best results of quickly making up ground between the two skiers (i.e., increasing Δ*x*) are achieved when the glide distances are long and the distance *D* between the skiers is short. Glide distances do not have to be necessarily straight and do not end at a corner. Long glide distances invite the skier to maintain a low tuck as long as possible, which in turn decreases Δ*x*. Therefore, slipstreaming at shorter glides and at higher tuck position also becomes advantageous as high tuck increases Δ*x*. At larger *D*, Δ*x* can become very ineffective, which is not of concern as it is anyway difficult to remain in the slipstream at larger *D*. The apparent disadvantage of the trailing skier's mass being smaller than the one of the leading skier is actually an advantage, as slipstreaming still reduces the effect of the difference mass makes without slipstreaming. When slipstreaming, it is essential to have at least the same degree of tuck as the leading skier. If the body height of the trailing skier is greater than the one of the leading skier, then a tucked position lower than the one of the leading skier is advantageous for being perfectly in the slipstream. If the leading skier lowers his/her tucked position, the trailing skier has to follow in order to make most out of the slipstreaming principles. There is no direct advantage of slipstreaming at higher velocity differential, i.e., if the speed of trailing skier is substantially higher than the one of the leading skier. Although a trailing skier's speed smaller than the one of the leading skier seems disadvantageous, slipstreaming is still advantageous as it reduces the speed loss.

When the distance *D* between the two skiers closes to a safe minimum before colliding, overtaking is the logical consequence. The suddenly increasing drag force when breaking out of the slipstream does not slow down the trailing skier. The trailing skier is still faster than the leading one, and the increased drag force merely reduces the acceleration of the trailing skier slightly. The trailing skier is still accelerating due to gravity. Overtaking is of advantage immediately before, and on the inner side of, a corner.

**Figure 3:**
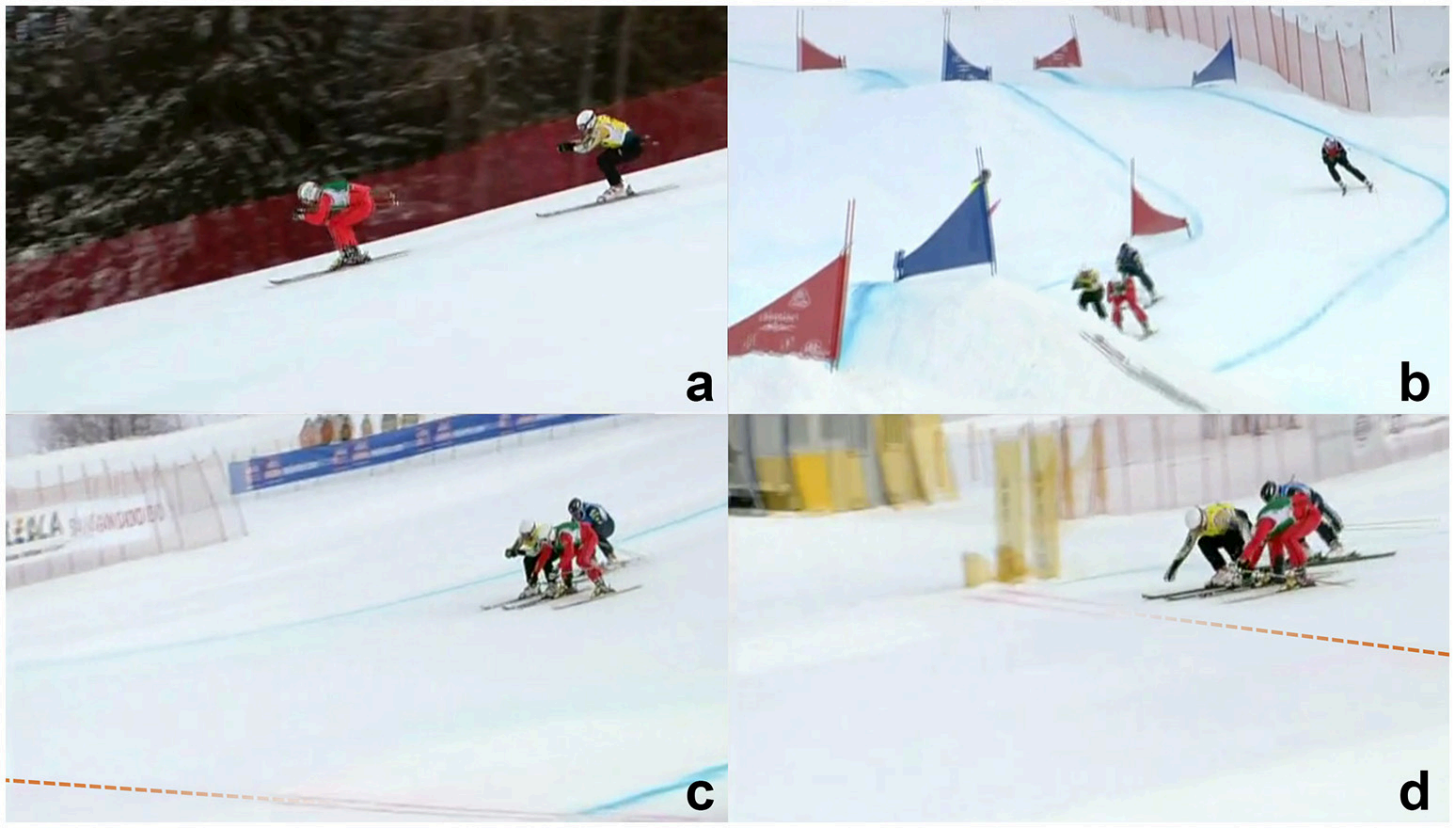
Example of slipstreaming and overtaking in ski cross; FIS Freestyle World Cup event in Innichen—San Candido on December 19, 2010; **(a)** Scott Kneller (yellow) slipstreaming about 4 m behind Alex Fiva (red); **(b)** Kneller breaks out of the slipstream on the inner side of the last corner before the last jump; **(c)** shortly before the finish line (dashed), Kneller has already overtaken Fiva and maintains his aerodynamic position whereas Fiva has already opened up; **(d)** immediately before the finish line, Kneller is clearly leading; video screenshots from: https://vimeo.com/18009110, © Konrad Rotermund 2010, reproduced with kind permission.

A classical example of slipstreaming over a long distance is given by the FIS Freestyle World Cup event in Innichen—San Candido on December 19, 2010 (Figure 3), which Scott Kneller (AUS) won 6 weeks after wind tunnel testing. Kneller slipstreamed behind Alex Fiva (SUI) for most of the race (Figure 3a) and broke out of the slipstream (Figure 3b) on the inner side of the last corner before the last jump (https://vimeo.com/18009110). He overtook the leading Alex Fiva (SUI) on the last slope before the finish line (Figure 3c), only to win by approximately 20 cm. Kneller maintained an advantageous aerodynamic position almost until the finish line, whereas Fiva opened up his arms and thereby experienced a higher drag force (Figure 3d).

The glide model presented in this research can be used in the future for testing of slope track design. There is some data on tracks available on the internet (e.g., https://wiki.fis-ski.com/index.php/Ski_Cross_Courses), however they are only related to major sections of the track in terms of length and slope angle. If more details were available, including precise dimensions of terrain features, the speed of one or even more skiers can be modeled and critical sections can be identified.

## Author contributions

The author confirms being the sole contributor of this work and approved it for publication.

### Conflict of interest statement

The author declares that the research was conducted in the absence of any commercial or financial relationships that could be construed as a potential conflict of interest.

## References

[B1] Barry N. Burton D. Sheridan J. Thompson M. Brown N. A. T. (2015). Aerodynamic drag interactions between cyclists in a team pursuit. Sports Eng. 18, 93–103. 10.1007/s12283-015-0172-8

[B2] Barry N. Sheridan J. Burton D. Brown N. A. T. (2014). The effect of spatial position on the aerodynamic interactions between cyclists. Proc. Eng. 72, 774–779. 10.1016/j.proeng.2014.06.131

[B3] Broker J. (1991). Model of Skiing Aerodynamics for the United States Olympic Committee. Colorado Springs, CO: United States Olympic Committee.

[B4] Drillis R. Contini R. (1966). Body Segment Parameters. Office of Vocational Rehabilitation. Department Health, Education & Welfare. BP174-945, Tech.Rep (1166-03), School of Engineering and Science, New York University New York, NY.

[B5] FIS (Fédération Internationale de Ski; International Ski Federation) (2017). The International Freestyle Skiing Competition Rules (ICR). Oberhofen: International Ski Federation FIS, 100 Available online at: http://www.fis-ski.com/mm/Document/documentlibrary/FreestyleSkiing/08/62/43/FS_FIS_FreestyleICR2017_English.pdf).

[B6] Fuss F. K. (2011). Wind Tunnel Tests on Slipstreaming in Skiing; Report for the Winter Olympic Institute of Australia. Melbourne, VIC: RMIT University.

[B7] Hoerner S. F. (1965). Fluid-Dynamic Drag. Bakersfield, CA: Hoerner Fluid Dynamics.

[B8] Kirby R. (2009). Development of a real-time performance measurement and feedback system for alpine skiers. Sports Technol. 2, 43–52. 10.1002/jst.85

[B9] Luethi S. M. Denoth J. (1987). The influence of aerodynamic and anthropometric factors on speed in skiing. Intl. J. Sport Biomech. 3, 345–352.

[B10] Nørstrud H. (2008a). Cross Country skiing, in Sports Aerodynamics, ed Nørstrud H. (Vienna: Springer), 107–130.

[B11] Nørstrud H. (2008b). Alpine downhill and speed skiing, in Sports Aerodynamics, ed Nørstrud H. (Vienna: Springer), 131–138.

[B12] Ortega J. O. Lindstedt S. L. Nelson F. E. Jubrias S. A. Kushmerick M. J. Conley K. E. (2015). Muscle force, work and cost: a novel technique to revisit the Fenn effect. J. Exper. Biol. 218, 2075–2082. 10.1242/jeb.114512 25964423 PMC6514452

[B13] Pitcher A. B. (2009). Optimal strategies for a two-runner model of middle-distance running. SIAM J. Appl. Math. 70, 1032–1046. 10.1137/090749384

[B14] Rundell K. W. (1996). Effects of drafting during short-track speed skating. Med. Sci. Sports Exerc. 28, 765–771. 8784765 10.1097/00005768-199606000-00016

